# Erythrocyte P2X_1_ receptor expression is correlated with change in haematocrit in patients admitted to the ICU with blood pathogen-positive sepsis

**DOI:** 10.1186/s13054-018-2100-3

**Published:** 2018-08-02

**Authors:** Steen K. Fagerberg, Parth Patel, Lars W. Andersen, Xiaowen Lui, Michael W. Donnino, Helle A. Praetorius

**Affiliations:** 1 Department of Biomedicine, Physiology, Aarhus University, Ole Worms Alle 3, build 1170, 8000 Aarhus C, Denmark; 2 Department of Emergency Medicine, Beth Israel Deaconess Medical Center, Harvard Medical School, Boston, MA USA; 3 Research Center for Emergency Medicine, Aarhus University Hospital, Aarhus, Denmark

**Keywords:** Sepsis, P2X, Toxins, Haemolysis, Anaemia, Exotoxemia/endotoxemia, Purinergic signalling, Haematocrit, Haemoglobin

## Abstract

**Background:**

Pore-forming proteins released from bacteria or formed as result of complement activation are known to produce severe cell damage. Inhibition of purinergic P2X receptors markedly reduces damage inflicted by cytolytic bacterial toxin and after complement activation in both erythrocytes and monocytes. P2X expression generally shows variation throughout the population. Here, we investigate correlation between P2X receptor abundance in blood cell plasma membranes and haematocrit during sepsis, in patients admitted to the emergency department (ED) or intensive care unit (ICU).

**Method:**

Patients admitted to the ED and successively transferred to ICU with the diagnosis sepsis (< 2 systemic inflammatory response syndrome (SIRS) criteria and suspected infection), were grouped as either blood pathogen-positive (14 patients) or blood pathogen-negative (20 patients). Blood samples drawn at ICU admission were analysed for P2X_1_ and P2X_7_ receptor abundance using indirect flow cytometry.

**Results:**

Here, we find inverse correlation between P2X_1_ receptor expression and change in haematocrit (*r*_s_ − 0.80) and haemoglobin (*r*_s_ − 0.78) levels from admission to ED to arrival at ICU in patients with pathogen-positive sepsis. This correlation was not found in patients without confirmed bacteraemia. Patients with high P2X_1_ expression had a significantly greater change in both haematocrit (− 0.59 ± 0.36) and haemoglobin levels (− 0.182 ± 0.038 mg/dl) per hour, during the first hours after hospital admission compared to patients with low P2X_1_ expression (0.007 ± 0.182 and − 0.020 ± 0.058 mg/dl, respectively).

**Conclusion:**

High levels of P2X_1_ are correlated with more pronounced reduction in haematocrit and haemoglobin in patients with confirmed bacteraemia. This supports previous in vitro findings of P2X activation as a significant component in cell damage caused by pore-forming bacterial toxins and complement-dependent major attack complex. These data suggest a new potential target for future therapeutics in initial stages of sepsis.

**Electronic supplementary material:**

The online version of this article (10.1186/s13054-018-2100-3) contains supplementary material, which is available to authorized users.

## Background

Anaemia is commonly observed in septic patients. There are several factors that contribute to acutely reduce the haemoglobin concentration during sepsis. Manifest systemic inflammation directly reduces the number of new erythrocytes introduced into the circulation (for review see [1]), whereas circulating bacteria and complement activation during sepsis can inflict erythrocyte damage that either result in the removal of the erythrocytes from the circulation or intravascular haemolysis. Preserving a high number of circulating erythrocytes is crucial for the oxygenation of the body during critical illness [2, 3] and interestingly, anaemia and wide erythrocyte distribution width are known to be independent predictors of death in septic patients [4]. These data fit a previous study demonstrating that a high level of free haemoglobin in the blood from patients admitted to the hospital with sepsis is correlated with a worse outcome [5].

**Fig. 1:**
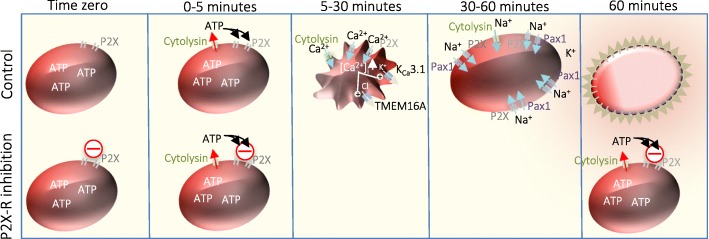
Model of pore former induced lysis. A bacterial toxin inserts a large channel or pore into the erythrocyte membrane. ATP is immediately released through the pore and activates P2X receptors. The membrane insertion of the toxin also causes a steep rise in the intracellular Ca^2+^ concentration ([Ca^2+^]_i_), which results from Ca^2+^ passing through the pore itself and from activation of P2X receptors, which are non-selective cation channels permeable to Ca^2+^. The increase in [Ca^2+^]_i_ activates the Ca^2+^-sensitive K^+^ channel K_Ca_3.1 and Cl^−^ channel TMEM16A, which results in K^+^ and Cl^−^ efflux and cell shrinkage as obligated water follows. The cells will remain shrunken as long as the K^+^ efflux surpasses the Na^+^ influx via the toxin pore and the P2X receptors. Prolonged stimulation of P2X_7_ can activate pannexins, which will contribute to the Na^+^ influx. Eventually, the Na^+^ influx will exceed the K^+^ efflux and the cells will swell and eventually burst. Blockage of the P2X_1_ and P2X_7_ receptor has been proven as a protective measure for bacterial toxins, and for complement to carry out their toxicity. The model based on previous work [6, 14, 17, 30]

As previously demonstrated, cell damage inflicted by several bacterial pore-forming toxins and complement-induced haemolysis is completely dependent on extracellular ATP-signalling [6–8]. If ATP-sensitive P2X receptors (P2X_1_ and P2X_7_) are blocked, it is possible to completely prevent cytolysin-induced haemolysis [6–10]. Thus, cytolysins such as α-haemolysin (HlyA) from *Escherichia coli* [6], α-toxin from *Staphylococcus aureus* [7], Apia haemolysin from *Actinobacillus pleuropneumoniae* [11], β-toxin from *Clostridium perfringens* [12]*,* leukotoxin (LtxA) from *Aggregatibacter actinomycetemcomitans* [8] and complement-dependent major attack complex [10] cause ATP release to the extracellular phase directly after membrane insertion of the pore [13]. A model of cytolysin-induced erythrocyte damage is illustrated in Fig. 1. Subsequent to pore insertion, the released ATP activates ligand-gated, ATP-sensitive P2X receptors, which are non-selective cation channels permeable to Ca^2+^ and Na^+^. Thus, pore insertion increases the intracellular Ca^2+^ concentration, which causse K^+^ and Cl^−^ efflux via Ca^2+^ sensitive channels (K_Ca_3.1 and TMEM16A) [14] leading to cell shrinkage [14]. Subsequently, the driving force for K^+^ exit diminishes and will be surpassed by Na^+^ influx via the toxin pore and P2X channels and the cell will swell and lyse. The Na^+^ influx is further supported by late activation of pannexin channels [6, 14]. Notably, toxin/complement-induced haemolysis does not happen instantaneously but is a protracted process of cell shrinkage and swelling, which is seen in vivo in erythrocytes during sepsis [15]. Increase in intracellular Ca^2+^ and cell shrinkage trigger exposure of phosphatidyl serine (PS) in the outer leaflet of erythrocytes. PS exposure is a strong signal for damaged erythrocytes to be recognised by phagocytotic cells (monocytes/macrophages) and removed from the bloodstream [16]. In vitro, blockage of P2X_1_ and P2X_7_ receptors abolishes both PS exposure and removal of the erythrocytes by monocytes [17] and thus, P2X receptors may potentially influence the number of circulating erythrocytes during sepsis. Thus, we hypothesise that high expression of P2X_1_ or P2X_7_ in the erythrocyte membrane may affect the number of circulating erythrocytes during sepsis. Moreover, since vitamin D has been associated with anaemia and is a regulator of cytokines in the immune response, we also measured vitamin D in the sample population.

Here, we in a small sample of septic patients demonstrate a clear reduction in haematocrit and haemoglobin from the arrival at the emergency unit to the admission to the special care unit. Strikingly, there is correlation between the reduction in both haematocrit and haemoglobin and the P2X_1_ receptor expression in the erythrocyte membrane from patients with confirmed bacteraemia. This correlation is not found in culture negative patients and potentially suggests an important function of the P2X_1_ receptor during sepsis.

## Methods

### Study design

This study was based on a convenience sample of septic patients admitted to the ICU from the ED at one tertiary care centre. Sepsis was defined as suspected infection based on at least two systemic inflammatory response syndrome (SIRS) criteria. Exclusion criteria were (1) known hereditary or malignant blood disease, (2) history of anaemia, (3) admission after trauma, or (4) known or ongoing visceral haemorrhage. Patients were grouped into a blood pathogen-positive and a blood pathogen-negative group based on blood culture results from the microbiology laboratory. Patients were further sub-grouped according to the bacterial capability of producing pore-forming toxins (the microbiologic profile in the pathogen-positive group is given in Table 1). It must be noted, however, that α-toxin from *S. aureus* requires very high concentrations to inflict damage on human erythrocytes because these cells lack the metalloprotease, a disintegrin and metalloproteinase with thrombospondin motifs (ADAM)10, which increases membrane insertion by adhesion of α-toxin to the membrane [18].

**Table 1:** Bacterial origin

Patient ID	Bacterial strain in blood	Toxin secretion	Toxin capable of haemolysis	P2X-dependent toxicity in vitro
#1	*Escherichia coli*	Yes	Yes, α-haemolysin	Yes
#2	Methcillin-resistant *Staphylococcus aureus*	Yes	Yes, α-toxin	Yes
#3	*Escherichia coli*	Yes	Yes, α-haemolysin	Yes
#4	*Staphylococcus aureus*, coagulase positive	Yes	Yes, α-toxin	Yes
#5	*Staphylococcus aureus*, coagulase negative	Yes	No	Unknown
#6	*Streptococcus anginosus* (Miller) group	Yes.	Yes	Unknown
#7	*Bacterioides fragilis*	Yes.	No	Unknown
#8	*Streptococcus anginosus* (Miller) group	Yes.	Yes	Unknown
#9	*Escherichia coli*	Yes	Yes, α-hemolysin	Yes
#10	*Clostridium difficile* (toxiogenic)	Yes	Yes, β-toxin	Inconclusive
#11	*Escherichia coli*	Yes	Yes, α-haemolysin	Yes
#12	*Escherichia coli*	Yes	Yes, α-haemolysin	Yes
#13	*Aeromonas hydrophila*	Yes	Yes,	Unknown
#14	*Candida* (*torulopsis*) *Glabrata*	Yes	Unknown	Unknown

Blood culture-positive patients and the pathogen found in the blood stream during microbiology blood sample examination, and the characteristics of their respective toxins

Blood samples from patients were drawn at admission and immediately stored at − 80 °C for experimental analysis. For detailed selection, see flowchart (Additional file 1). The study was approved by the Institutional Review Board and written informed consent was obtained prior to enrolment.

### Data handling

All data were collected by a trained research assistant according to a detailed, pre-defined data dictionary. Data were entered into a secure, online database (Research Electronic Data Capture (RedCAP)).

### Haematocrit and haemoglobin assessment

Haematocrit and haemoglobin levels were extracted from blood samples drawn at Emergency Department (ED) admission, ICU admission and after 24 and 48 h of ICU admission, as registered in RedCAP. The difference in haematocrit and haemoglobin values between those obtained in the ED and in the ICU, 24 h after admission and 48 h after admission, was divided by the time in minutes between the measurements. Haematocrit was measured as the fraction of erythrocytes in the whole blood samples in percent and haemoglobin was measured as mg/dl. The rate of change in values is presented as Δ%/hour for haematocrit and Δ(mg/dl)/hour for haemoglobin with a positive value indicating an increase.

### Patient material

Blood samples stored at − 80 °C as whole blood samples at the Center for Resuscitation Science, BIDMC, Boston, USA. Samples were drawn between 0 and 24 h after ICU admission. Whole blood samples were quickly thawed in a 37 °C water bath. Since the erythrocytes samples were not frozen in glycerol, defrosting was expected to cause haemolysis. Blood samples with no visible erythrocyte pellet after thawing were excluded. Thawed blood samples were immediately centrifuged at 300 *g* for 10 min, to remove free haemoglobin. Remaining cells together with membrane from the lysed cells were diluted in HEPES-buffered salt solution (HBS) and centrifuged twice at 600 *g* for 3 min followed by removal of plasma and/or supernatant. Erythrocytes were diluted to 1% *v*/v, or approximately 1.75 × 10^6^ cells/ml. All blood samples were inspected under a light microscope before further handling. It was noted that all blood samples in addition to healthy-looking erythrocytes, had sub-populations of both crenated and swollen erythrocytes due to the freezing-thawing procedure.

### P2X receptor quantification

Cells in each sample were counted on a cell counter (Spectre, Merch Millipore, USA) prior to exposure to either P2X_1_ (catalogue number APR-022-AG) or P2X_7_ (catalogue number APR-008-F) fluorescein isothiocyanate (FITC)-conjugated antibody (Alomone Labs, Jerusalem, Israel) for 1 h at room temperature in the dark at 200 rpm in concentrations according to the manufacturer’s instructions (10 mg antibody to 10^6^ cells). The P2X receptor expression was quantified by flow cytometry (Gallios, Beckman, Indianapolis, USA) available through the Center of Life Science, BIDMC, and based on antibody fluorescence excitation at 488 nm. Control peptide provided by Alomone Labs was added to the cell suspension of five randomly selected patients before the addition of the labelled antibodies for comparison. The flow cytometer was adjusted to exclude background noise and erythrocyte membrane debris without excluding damaged erythrocytes. This was done by a series of test experiments applying phosphate-buffered salt solution (PBS) alone and isolated washed erythrocytes only, to identify background noise and location of the erythrocyte population. Erythrocytes were identified based on side scatter (SSC) and forward scatter (FSC), and geometric mean fluorescence intensity (gMFI) was measured in standardised regions of interest (ROIs). Regions were chosen based on existing knowledge on the range interval in FSC and SSC following a freeze-thaw cycle, which increases the range of both. All samples of washed erythrocytes contained populations of cells within the FSC/SSC ranges that correspond to monocytes and granulocytes/macrophages. Each blood sample was measured for background fluorescence using the same setup, in the absence of labelled antibody or control antibody. Samples compared in statistical correlation tests were exposed to the same stock of antibody, chemicals and solutions, and sample fluorescence was measured during same experiment cycle, within the same 30-min period.

### Measurement of vitamin D

The vitamin D enzyme-linked immunosorbent assay (ELISA) kit from Cayman chemical was used to measure vitamin D. Patient plasma stored at − 80 °C was thawed and added to a rabbit polyclonal IgG anti-sheep-coated well plate. Vitamin D concentration was assessed based on the reaction between vitamin D and an AChE conjugate (vitamin D tracer). After this reaction, absorption was measured at 412 nm by photo-spectrometry and quantified using standard curves provided by the manufacturer.

### Immunoblot experiments for method validation

Blood samples drawn from healthy volunteers were isolated, lysed and washed four times with 10 mM Tris solution. After each wash, the suspension was spun (16,000 *g,* 30 min, 4 °C) and the haemoglobin-containing supernatant carefully removed. The final pellet was dissolved in Tris-solution, separated by electrophoresis on 12% mini-protean TGX precast gel (Bio-Rad) and blotted onto a polyvinylidene fluoride (PVDF) membrane (Thermo Scientific). Unspecific binding was reduced by 1% bovine serum albumin powder in PBS, overnight at 4 °C. The membranes were washed and incubated overnight at 4 °C with the same primary antibody as used for flow cytometry diluted in PBS with 0.1% Tween20. Pre-adsorption controls were included for all antibodies with 1:1 peptide-antibody ratio. The membranes were washed thoroughly in PBS-Tween and incubated with peroxidase conjugated anti-rabbit Ig antibody (DAKO, Glostrup, Denmark). Excess antibody was removed by extensive washing in PBS-Tween, and bound antibody was detected by ClarityTM Western ECL substrate (Bio-Rad) and bands visualised in a Quant LAS mini system (GE Healthcare Life Science, Pittsburg, PA, USA). Protein content was determined for isolated erythrocyte membranes using BCA Protein Assay Kit (Thermo Scientific). The FITC-conjugated primary antibody used for flow cytometry quantification could not be used directly in immunoblotting because of a low fluorescence signal. To obtain a sufficient signal, we applied a secondary HRP-conjugated rabbit anti-goat antibody for visualisation. These experiments were carried out at the Department of Biomedicine, Aarhus University, Denmark. Representative immunoblots are included in Additional file 2.

### Freeze-thaw control experiments

Blood from healthy volunteers was drawn from volunteers according to permission given by Danish Scientific Ethics Committee (M20110217). A small sample was diluted 1000-fold and tested for cell population and background haemolysis. The remaining whole blood sample was centrifuged at 300 *g* for 5 min and vehicle or P2X_1_ and P2X_7_ antagonist was added to the plasma phase to secure proper dilution. The whole blood sample was now re-suspended and incubated at 37 °C at constant swirl (150 rpm) for 15 min, after which the blood sample was stored at − 80 °C for 2 weeks. Hereafter samples were thawed according to the protocol from septic blood samples, and haemolysis and cell population distribution were measured. These experiments were carried out at Department of Biomedicine, Aarhus University, Denmark.

### Material and solutions

We used HBS in mM: [Na^+^] 138.0, [Cl^−^] 132.9, [K^+^] 5.3, [Ca^2+^] 1.8, [Mg^2+^] 0.8, [SO_4_^2−^] 0.8, [HEPES] 14, [glucose] 5.6, pH 7.4 at 37 °C; PBS in mM: [Na^+^] 156.9, [Cl^−^] 139.6, [K^+^] 4.4, [HPO_4_^2−^] 10, [H_2_PO_4_^−^] 1.8 (for PBS-Tween, 1 ml Tween is added per litre)*.* FITC-conjugated antibodies directed against the extracellular loop of P2X receptor were purchased form Alomone, Jerusalem, Israel and NF449 and A804598 was supplied by Tocris, Bioscience, Bristol, UK.

### Statistical analysis

Descriptive statistics were provided as means or medians with standard deviations (SD) or 1st and 3rd quartiles. Continuous data were compared between groups using the Student *t* test for normally distributed data and Wilcoxon rank sum test for data that were not normally distributed. Correlation was assessed by calculating Spearman’s correlation coefficient (*r*_s_). All hypothesis tests were two-sided, with a significance level of *p* < 0.05. Statistical analyses were performed with the use of Prism Software, version 6.

## Results

### Characterisation of the patient demographics and clinical parameters

**Fig. 2:**
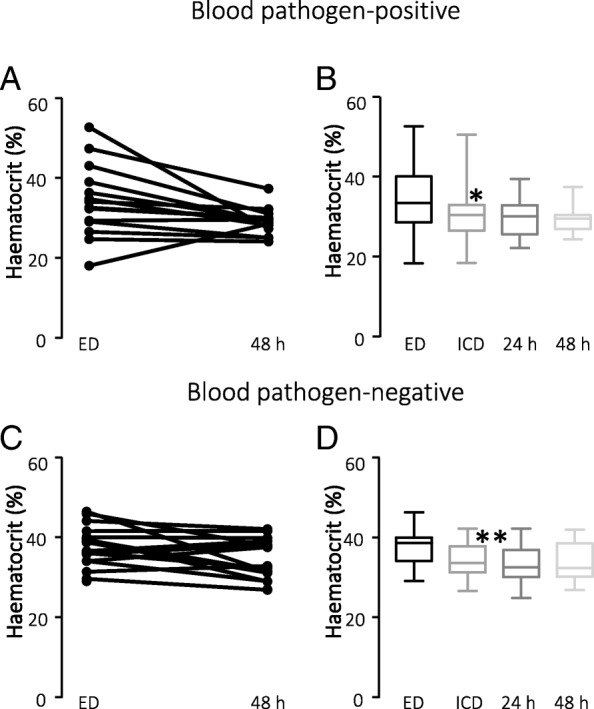
Changes in haematocrit in sample populations. **a** Change in haematocrit in patients with confirmed pathogens in the blood from admission to the Emergency Department (ED) until 48 h after admission. **b** Mean values of haematocrit in the same patient sample as in **a** at ER admission, ICU admission, 24 h after ICU admission and 48 h after ICU admission. **c** Change in haematocrit in patients without confirmed pathogens in the blood from admission to the ED until 48 h after admission. **d** Mean values of haematocrit in the same patient sample as in **c**

Our final study group consisted of 14 patients in the pathogen-positive group and 20 patients in the pathogen-negative group. The two groups of patients admitted under suspicion of sepsis - the blood pathogen-positive (*n* = 14) and blood pathogen-negative (*n* = 20) groups - had similar characteristics with regards to age, length of hospitalisation, etc. (see Table 2). Patient vital signs (blood pressure, pulse, respiration rate and temperature) were not statistically significantly different between the two groups; however, patients suffering from sepsis confirmed by a positive blood culture result had a markedly higher heart rate (*p* < 0.05, see Table 2). The mortality rate was not statistically significantly different between the two groups (for further details see Table 2). The change in haematocrit was most significant within the first hours after the hospital admission, reaching maximum in both groups between ED and ICU admission, and any development in haematocrit after ICU admission was not statistically significantly different (Fig. 2b, d). The velocity of the decrease in haematocrit was highest in both groups between ED and ICU admission (Fig. 2a, c). Since vitamin D levels previously have been speculated to influence the outcome of severe sepsis and have been linked to anaemia [19], we measured vitamin D (25-OH Vit D3) levels in the two groups and found them similar with a median value of 26.12 ng/ml in the pathogen-positive group and 23.35 ng/ml in the pathogen-negative group.

**Table 2:** Paraclinical data

Demographics	Culture positive (*n* = 14)	Culture negative (*n* = 20)
Age, year	65.5 (59.2–71)	71 (65.5–78)
Weight, kg	82.95 (71.4–89.8)	86.1 (68.9–99.8)
Sex, female, *n* (%)	6 (43)	5 (20)
Race, *n* (%)		
-White	14 (100)	18 (90)
-Black	0 (0)	1 (5)
-Other/not specified	0 (0)	1 (5)
Time between blood samples from ER and ICU	11.1 (6.6–13.3)	9.7 (7.4–17.5)
Time from admission to ER and to ICU	2.7 (1.9–3.5)	2.3 (1.7–5.12)
length of stay in ICU, median (days)	4.4 (1.0–6.75)	3.6 (1.25–4.65)
Length of stay in hospital, median (days)	11.6 (6.25–13.75)	10.5 (6.25–11.75)
Laboratory values at admission to ER, median		
-Hct, median, %	33.6 (29.4–39.2)	36.8 (34.5–38)
-Hgb	10.7 (9.6–12.9)	12.5 (11.4–13.5)
-Monocyte count	5.7 (3.3–8.0)	3.5 (3.0–5.3)
-White blood cell count	13.3 (7.8–14.2)	13.4 (9.6–15.8)
-Platelet count	283.5 (157.8–367)	181.5 (135.0–246.8)
-Lactate	2.3 (1.6–3.1)	1.9 (1.2–2.4)
-MCV	97 (89.5–102)	90 (87.3–93.5)
Laboratory values at admission to ICU, median		
-Hct	30.5 (27.8–31.9)	34.5 (31.6–37.6)
-Hgb	9.7 (8.8–10.8)	11.3 (10.2–12.4)
-Monocyte count	4.6 (3.3–5.6)	6 (2.2–7.3)
-White blood cell count	11.9 (7.2–18)	11.4 (8.1–14.0)
-Neutrophil granulocytes	77.8 (69.5–79.8)	82.5 (79.6–89.8)
-Platelet count	215.5 (131.3–325)	167 (111.8–234.5)
-Lactate	2.6 (1.1–3.1)	1.8 (1.1–2.4)
-MCV	97.5 (90.3–103)	90.5 (85–94.3)
-Vitamin D	26.12 (17.22–32.13)	23.35 (17.75–27.34)
Vital signs at ICU admission		
-Heart rate, median, bpm	111 (88–118)	92 (78.8–106.5))
-Respiratory rate, median, rpm	20 (17.5–22)	20 (18.5–25.3)
-Temperature, median, F	99.5 (98–100)	98.7 (98.1–99.1)
-SBP, median, mmHg	90.5 (83.3–97)	88 (78.8–93.2)
-DBP, median, mmHg	47 (43.3–56)	44.4 (36.5–48)
-Saturation, median, % oxygen	97 (96–99)	96.5 (93.8–100)
Outcome, *n* (%)		
-Home/home with service	6 (43)	11 (55)
-Rehabilitation/nursing home	7 (50)	7 (35)
-Deceased	1 (7)	2 (10)

### Characterisation of the patient P2X expression

Within the two patient populations, we found no statistically significant difference in the distribution of the erythrocyte P2X_1_ receptors with an expression level of 5.0 (3.4; 6.0) in the blood pathogen-positive group and 5.1 (3.6; 6.8) in the blood pathogen-negative group. There was, however, slightly but statistically significantly lower expression of P2X_7_ receptors in the blood pathogen-positive group (*p* < 0.05). With regards to monocyte expression of P2X receptors, patients in the blood pathogen-positive group had lower P2X_1_ expression (*p* = 0.02) but higher P2X_7_ expression (*p* = 0.05). This observation potentially supports previous findings suggesting pathogen-induced up-regulation of the P2X_7_ receptors on human monocytes [20]. This picture was reversed in granulocytes from the blood pathogen-positive patients, in whom the P2X_1_ receptor expression was higher compared to the pathogen-negative patients (*p* < 0.0001, for further information see Table 3). There was no correlation between the amount of either P2X_1_ or P2X_7_ receptor and vital parameters or vitamin D levels.

**Table 3:**
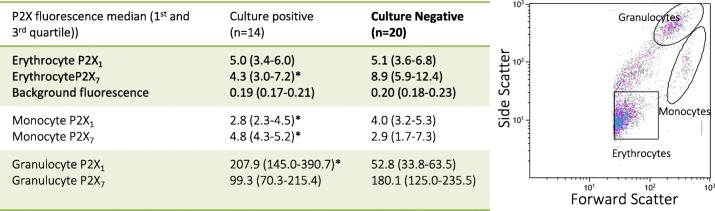
Flow cytometry

Detection of P2X receptors on erythrocytes. A) The table shows average receptor expression within patient groups. Values are given as medians with 1st and 3rd quartile in brackets. B) shows the flow cytometry gating for all experiments. *Indicates statistically significant difference between the groups

**Fig. 3:**
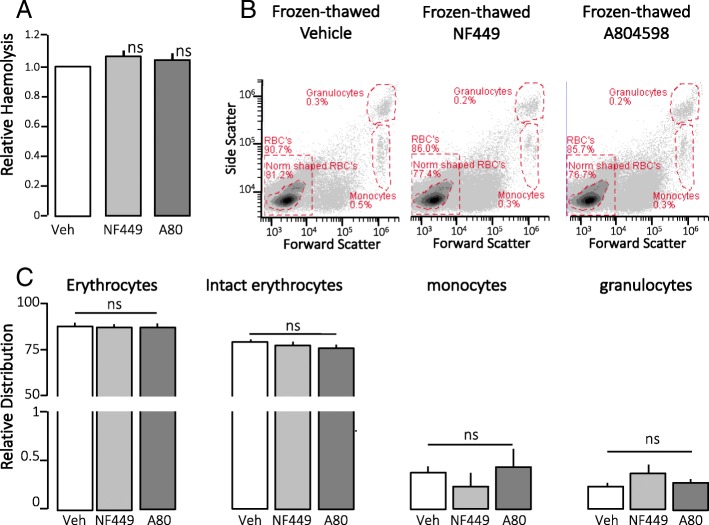
Freeze-thaw - control experiments. **a** Whole blood drawn from healthy volunteers was incubated either with vehicle (Veh), 100 μM NF449 (P2X_1_ antagonist) or 100 μM A804598 (P2X_7_ antagonist) and frozen and stored for 3 weeks as whole blood at − 80 °C. After thawing, lysis was measured as absorbance of free haemoglobin in supernatant at 540 nm. *n* = 3, mean ± SEM. **b** Flow cytometry of thawed whole blood samples diluted 1000-fold, to ascertain equal distribution of blood cell sub-populations. Cells were identified by forward scatter FSC and side scatter SSC and quantified based on fixed regions. **c** Bar graph shows data presented as mean ± SEM, *n* = 3. ns, not significant

P2X_1_ and P2X_7_ activation markedly amplify the haemolysis inflicted by HlyA [6]. Therefore, we tested P2X_1_ and P2X_7_ inhibition with the P2X_1_ receptor antagonist NF449 and the P2X_7_ receptor antagonist A804598, at concentrations known to completely block HlyA-induced haemolysis. Please note that inhibition of P2X receptors did not inhibit the haemolysis after freezing and thawing of erythrocytes (Fig. 3).

### P2X_1_ receptor expression on erythrocytes and change in haematocrit and haemoglobin

**Fig. 4:**
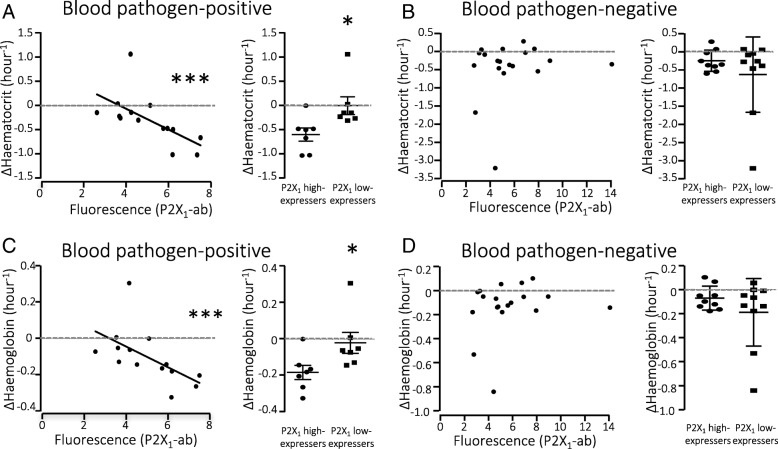
Haematocrit and haemoglobin levels and erythrocyte P2X_1_ receptor expression. **a** Change in haematocrit (hct) in blood pathogen-positive patients with sepsis, between Emergency Department (ER) and ICU admissions and P2X_1_ receptor expression on erythrocytes. The right panel shows blood pathogen-positive patients grouped by high or low expression of P2X_1_ (Δhct/hour, *p* = 0.011). **b** Change in haematocrit in blood pathogen-negative patients with sepsis between ER and ICU admission was not correlated with P2X_1_ receptor expression on erythrocytes. The right panel shows erythrocyte turnover rate (Δhct/hour) in high P2X_1_-expressing or low P2X_1_-expressing patients. **c** Change in haemoglobin in blood pathogen-positive patients with sepsis between ER and ICU admissions and P2X_1_ receptor expression on erythrocytes. The right panel shows the change in haemoglobin (ΔHgb/hour) in blood pathogen-positive patients grouped by high or low expression of P2X_1_ (*p* = 0.0375). **d** Change in haemoglobin from ER-admission to ICU in blood pathogen-negative patients with sepsis and P2X_1_ receptor expression on erythrocytes. The right panel shows change in haemoglobin (ΔHgb/hour) between high P2X_1_-expressing and low P2X_1_-expressing patients. Data presented as mean ± SEM

By examining the surface expression of the P2X_1_ receptors on erythrocytes, we found inverse correlation between the degree of receptor expression and the change in haematocrit between the blood sample drawn at ED admission and the first from ICU, with *r*_s_ − 0.80 (CI − 0.94; − 0.45, *p* = 0.001, Fig. 4a) (Table 4). This was not the case within the blood pathogen-negative patients (Fig. 4b). We applied the same correlation test and grouping to the change in haemoglobin, and found similar inverse correlation with *r*_s_ − 0.78 (CI − 0.93; − 0.41, *p* = 0.0015, Fig. 4c) in the blood pathogen-positive patient group, again without correlation in the blood pathogen-negative group (Fig. 4d). Importantly, we did not observe any correlation between the P2X_1_ or P2X_7_ receptor expression and the volume of intravenous fluid given to the patients, either in the control (P2X_1_ *r*_s_ = − 0.29, *p* = 0.2096; P2X_7_ *r*_s_ = 0.03, *p* = 0.9148) or in the pathogen-positive group (P2X_1_ *r*_s_ = − 0.08, *p* = 0.7966; P2X_7_ *r*_s_ = 0.39, *p* = 0.1736. This means that the change in haemoglobin cannot be explained by dilution of circulating erythrocytes.

**Table 4:** Correlation statistics

	P2X dependant toxin activity	Haemolytic activity	Pathogen-positive	Pathogen-negative
Correlation	Spearman *r* (confidence interval), *n*, *p* value	Spearman *r* (confidence interval), *n*, *p* value	Spearman *r* (confidence interval), *n*, *p* value	Spearman *r* (confidence interval), n, *p* value
P2X_1_ expression and change in haematocrit	− 0.82 (exact), 7, 0.0341	− 0.85 (− 0.95 to − 0.51), 11, 0.0015	− 0.80 (− 0.93 to − 0.45), 14, 0.0010	0.19 (− 0.31 to 0.60), 20, 0.4459
P2X_1_ expression and change in haemoglobin	− 0.79 (exact), 7, 0.0480	− 0.81 (− 0.95 to − 0.39), 11, 0.0039	−0.78 (− 0.93 to − 0.41), 14, 0.0014	0.19 (− 0.30 to 0.61), 20, 0.4372
P2X_7_ expression and change in haematocrit	0.2143 (exact), 7, 0.4444	− 0.19 (− 0.72 to 0.48), 11, 0.5619	0..02 (− 0.53 to 0.56), 14, 0.9363	0.22 (− 0.28 to 0.62), 20, 0.3749
P2X_7_ expression and change in haemoglobin	0.2143 (exact), 7, 0.3024	− 0.18 (− 0.71 to 0.49), 11, 0.5894	0.02 (− 0.53 to 0.56), 14, 0.9363	0.03 (− 0.44 to 0.50), 20, 0.8922

Values are given as Spearman *r* with confidence interval in brackets., number of patients included, and *p* value

By grouping the blood pathogen-positive patients based on erythrocyte P2X_1_ expression, we saw a statistically significant difference in the haematocrit (ΔHct/hour) between high-expressing (> 5.0 fluorescence units) and low-expressing (< 5.0 fluorescence units) groups (*p* = 0.011, Fig. 4a, right panel). Patients with high P2X_1_ expression experienced a change in haematocrit of 0.59 ± 0.36 ΔHct/hour, whereas patients with low P2X_1_ expression experienced a change of 0.007 ± 0.182 (*p* = 0.011, Fig. 4a, right panel). This statistically significant difference was seen for haemoglobin levels as well, with an average change of − 0.182 ± 0.038 mg/dl in patients with high P2X_1_ expression and 0.020 ± 0.058 mg/dl in patients with low P2X_1_ expression.

### P2X-dependent toxicity of pore formers

**Fig. 5:**
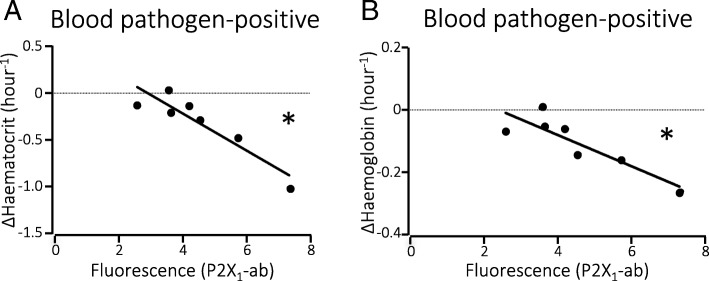
Haematocrit and haemoglobin levels and erythrocyte P2X receptor expression in patients infected with cytolysin-producing bacteria. **a** Change in haematocrit between Emergency Department (ER) and ICU admissions in patients with a positive blood culture result for cytolysin-producing bacteria and correlation with P2X_1_ receptor expression on erythrocytes. **b** Change in haemoglobin between ER and ICU admissions in patients with a positive blood culture result for cytolysin-producing bacteria and correlation with P2X_1_ receptor expression

When limiting the data to only include patients with sepsis caused by *E. coli* and *S. aureus,* known to have P2X-dependent toxicity, there was still negative correlation between P2X_1_ expression and the change in haematocrit and haemoglobin of *r*_s_ = − 0.82 (*p* = 0.034, *n* = 7, Fig. 5a) and *r*_s_ = − 0.79 (*p* = 0.048, n = 7, Fig. 5b), respectively. Looking only at the bacteria known to secrete toxins with haemolytic activity strengthened the correlation, with *r*_s_ = − 0.85 (CI − 0.96; − 0.51, *p* = 0.0015, *n* = 11) for haematocrit levels and exact *r*_s_ = − 0.81 (CI − 0.95; − 0.39, *p* = 0.0039, *n* = 11) for haemoglobin. No correlation was found between the non-haemolytic toxin-producing bacterial strains and change in haematocrit or haemoglobin levels.

### P2X_7_ receptor expression on erythrocytes and change in haematocrit and haemoglobin

**Fig. 6:**
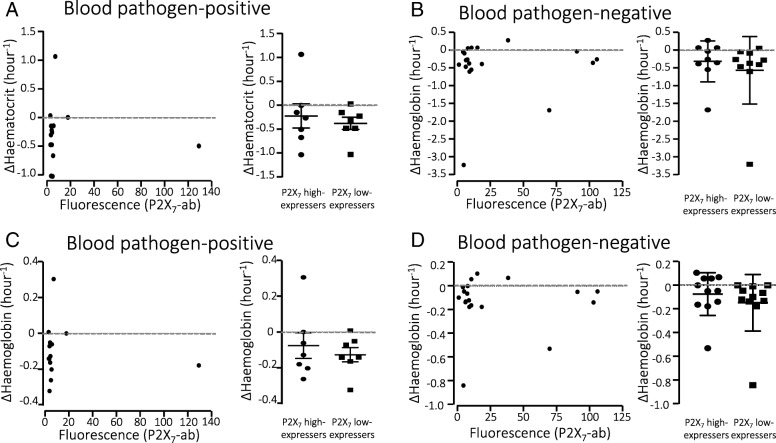
Haematocrit and haemoglobin levels and erythrocyte P2X_7_ receptor expression. **a** Change in haematocrit in blood pathogen-positive patients with sepsis between Emergency Department (ER) and ICU admission and P2X_7_ receptor expression on erythrocytes were not correlated. The right panel shows the change in haematocrit in blood pathogen-positive patients grouped by high or low expression of P2X_7_ (Δhct/hour). **b** Change in haematocrit in blood pathogen-negative patients with sepsis between ER and ICU admission and P2X_7_ receptor expression on erythrocytes. Right panel shows the change in haematocrit in blood pathogen-negative patients grouped by high or low expression of P2X_7_ (Δhct/hour). **c** Change in haemoglobin in blood pathogen-positive patients with sepsis between ER and ICU admission and P2X_7_ receptor expression on erythrocytes were not correlated. The right panel shows change in haemoglobin in blood pathogen-positive patients grouped by high or low expression of P2X_7_ (ΔHgb/hour, not significant (ns)). **d** Change in haemoglobin in blood pathogen-negative patients with sepsis between ER and ICU admission and P2X_7_ receptor expression on erythrocytes. The right panel shows change in haemoglobin in blood pathogen-negative patients grouped by high or low expression of P2X_7_ (ΔHgb/hour)

P2X_7_ receptors have also been demonstrated to amplify the in vitro lysis of erythrocytes by bacterial pore-forming toxins [6–8] and complement activation [10]. We did, however, not find any correlation between surface expression of P2X_7_ receptors and the change in haematocrit or haemoglobin between the blood sample drawn at ED admission and at ICU admission neither in the pathogen-positive (Fig. 6a, c) or the pathogen-negative patients (Fig. 6b, d). Grouping the blood pathogen-positive patients into low P2X_7_-expressing (< 4.3 fluorescence units) and high P2X_7_-expressing (> 4.3 fluorescence units) groups did not reveal any statistically significant difference in either ΔHct/hour or Δhaemoglobin/hour between the groups (Fig. 6a, b right panels). We found no correlation between the length of hospital stay, either in ICU or in total hospital stay, and P2X_1_ or P2X_7_ expression.

## Discussion

Our results show inverse correlation between the change in haematocrit and haemoglobin and the amount of P2X_1_ expression on the erythrocyte surface. This correlation is only found where a pathogen is present in the bloodstream, and only for P2X_1_ and not for P2X_7_. This inverse correlation was strengthened when only looking at pathogens capable of secreting bacterial toxins with haemolytic activity, suggesting that low P2X_1_ surface expression on the erythrocyte may be a protective attribute during bacteremia.

Sepsis is an overwhelming and life-threatening response to infectious agents and is the major cause of death in intensive care units worldwide. The condition, when untreated, often leads to multiple organ failure as a result of uncontrolled immune system activation with infiltrating immune cells and high levels of pro-inflammatory cytokines [21, 22]. The pathogens in the blood causing the immune activation are likely to inflict cell damage, which can result in the release of intracellular components such as ATP to the extracellular environment, where they have pro-inflammatory functions. Many of the bacteria that cause sepsis are capable of producing virulence factors, which have lytic properties. Moreover, activation of the complement system during sepsis may contribute to acute cell damage. Neither the cytolytic bacterial toxins nor the pore formed in major attack complex cause lysis immediately after they are inserted into the membrane [8, 10, 14]. The lytic process is protracted as illustrated in Fig. 1, with an initial shrinkage phase followed by slow swelling and eventually lysis [6–8, 10, 14, 23]. This consecutive shrinkage (crenation) of the erythrocytes can be observed directly in blood drawn from patients with sepsis [15]. The cells do not have to lyse to release ATP and ATP release through the pore is one of the earliest signs of a cell membrane insertion of the cytolysin [13]. Since ATP release is an implicit hallmark of cytolysin attack, it is striking that cytolysin-induced cell damage in vitro is completely prevented by inhibition of ATP-sensitive P2X receptors [6, 7, 10]. It must be stressed that inhibition of P2X receptor not only prevent lysis but also phosphatidylserine exposure and erythro-phagocytosis by monocytes and macrophages [17]. Thus, lysis and erythro-phagocytosis, two mechanisms potentially altering haematocrit, are P2X receptor-dependent in vitro*.*

Here, the patient group with confirmed bacteraemia had a marked reduction of haematocrit just after admission. This may reflect both haemolysis and removal of damaged erythrocytes from the bloodstream by phagocytes [24]. Interestingly, there was correlation between the number of P2X_1_ receptors expressed in the erythrocyte membrane and the reduction in both haematocrit and haemoglobin concentration. These results support the notion that P2X_1_ receptors are important for acute cell damage during severe infection. The majority of the pathogen-positive sample population were infected with a bacterial strain with haemolytic probabilities. Approximately half of this sample population were infected with bacteria known to secrete cytolysins, known to inflict early ATP release. We confirmed and strengthened the correlation between P2X_1_ receptor expression and reduction in haematocrit and haemoglobin in these sub-populations respectively. These findings additionally suggest that many other haemolytic toxins may have P2X-dependant properties as well.

Reduction in haemoglobin may potentially influence tissue oxygenation, which theoretically could be important for translation into septic shock. Previous studies have shown that both oxygen delivery and free haemoglobin are associated factors in mortality in sepsis [2, 3], and both of these parameters are dependent on erythrocyte membrane integrity. However, the effect of blood transfusions on increasing tissue oxygenation in the septic patient has been controversial. This effect may be lacking due to an additional challenge of transfusion-related syndromes, which is not the case when host erythrocytes are protected [25]. Thus, one could speculate that P2X_1_ receptor inhibition could be beneficial during severe infection. On this note, a recent study on sepsis induced by uro-pathogenic *E. coli*, showed that P2X_1_ deficient mice have reduced cytokine levels in the blood and a distinctly lower degree of intravascular coagulation in response to the infection compared to controls [26].

Despite the P2X_7_ receptor clearly being involved in cytolysin-induced cell damage, we did not find any correlation between the P2X_7_ receptor and any of the registered blood parameters. Pathogen-positive patients had markedly lower P2X_7_ receptor expression on the erythrocytes than the pathogen-negative patients. One could potentially speculate that patients with low P2X_7_ receptor expression are more prone to develop sepsis either due to inadequate innate immune response or other factors. There is marked single nucleotide polymorphism (SNP) viability of human P2X_7_ receptors (for review see [27]) and SNP variations in the P2X_7_ receptor has been associated with the susceptibility of tuberculosis [28] and toxoplasmosis [29].

Our results presented here are based on a small convenience sample enrolled at one centre, and thus may not be representative of a more complex patient group. Additionally, decreases in haematocrit based on dilution due to fluid treatment is hard to disregard in our calculations; however, this dilution factor is not likely to be dependent on the amount of P2X receptor expression and should thus affect both groups.

## Conclusion

Our data demonstrate clear correlation between P2X_1_ receptor expression on erythrocyte membranes and reduction in haematocrit and haemoglobin observed upon hospitalisation of patients with sepsis and confirmed bacteraemia. Despite the small sample size, our data support the notion that P2X_1_ receptor activation may reinforce a reduction in circulating erythrocytes during bacteraemia. Further studies with larger patient groups are needed in order increase the external validity of our findings.

## Additional files

Additional file 1: Patient selection flow chart. Schematic of selection of patients included in the study. (PDF 112 kb)

Additional file 2: Verification of antibodies. Full immunoblots for the P2X_1_ and P2X_7_ antibodies used for flow cytometric detection of the P2 receptors on the erythrocytes, with or without peptide pre-adsorption. The proteins used were isolated plasma membranes for human erythrocytes. (PDF 112 kb)

## Abbreviations

ED: Emergency Department
FSC: Forward scatter
Hbg: Haemoglobin
Hct: Haematocrit
PBS: Phosphate-buffered saline
PS: Phosphatidyl serine
RedCAP: Research Electronic Data Capture
SSC: Side scatter
SIRS: Systemic inflammatory response syndrome
