# Supplementary figures and images for: Erythrocyte P2X1 receptor expression is correlated with change in haematocrit in patients admitted to the ICU with blood pathogen-positive sepsis
Source: Crit Care. 2018 Aug 2;22:181. doi: 10.1186/s13054-018-2100-3 (PMC6091015; doi:10.1186/s13054-018-2100-3)

# Additional file 1

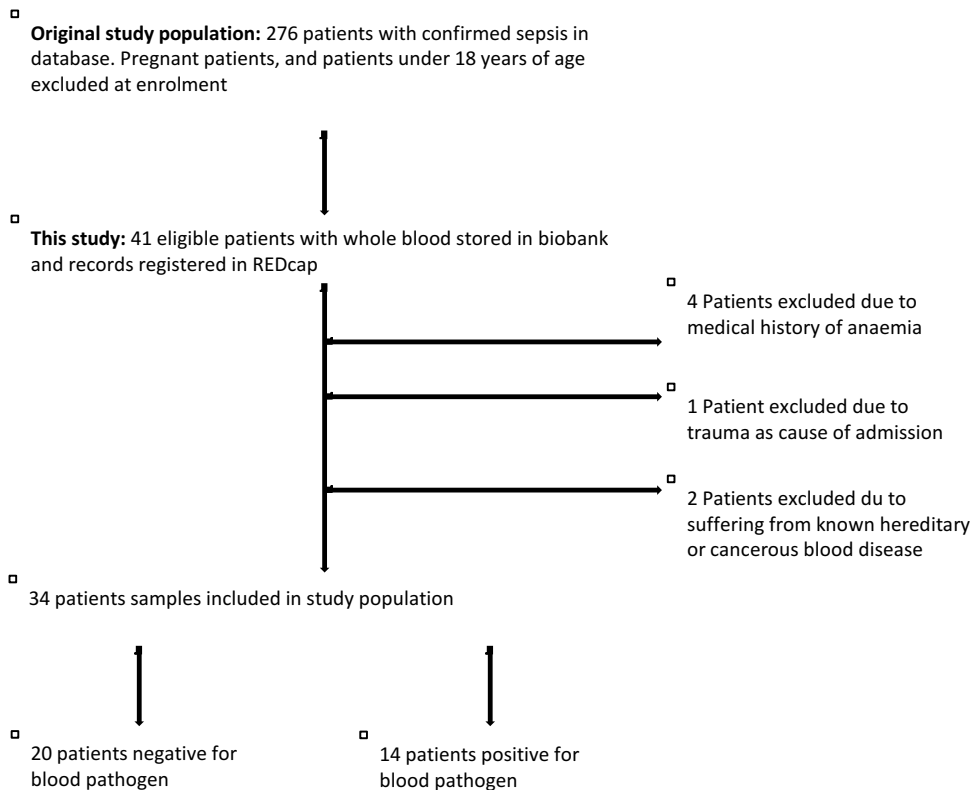

Additional file 1

Patient selection flow chart

Supplement: Supplementary file 1 — Patient selection flow chart. Schematic of selection of patients included in the study. (PDF 112 kb) [file 13054_2018_2100_MOESM1_ESM.pdf]
