# Supplementary material for: Erythrocyte P2X1 receptor expression is correlated with change in haematocrit in patients admitted to the ICU with blood pathogen-positive sepsis
Source: Crit Care. 2018 Aug 2;22:181. doi: 10.1186/s13054-018-2100-3 (PMC6091015; doi:10.1186/s13054-018-2100-3)
Supplement: Supplementary file 2 — Verification of antibodies. Full immunoblots for the P2X1 and P2X7 antibodies used for flow cytometric detection of the P2 receptors on the erythrocytes, with or without peptide pre-adsorption. The proteins used were isolated plasma membranes for human erythrocytes. (PDF 112 kb) [file 13054_2018_2100_MOESM2_ESM.pdf]

Additional file 2-verification of antibodies

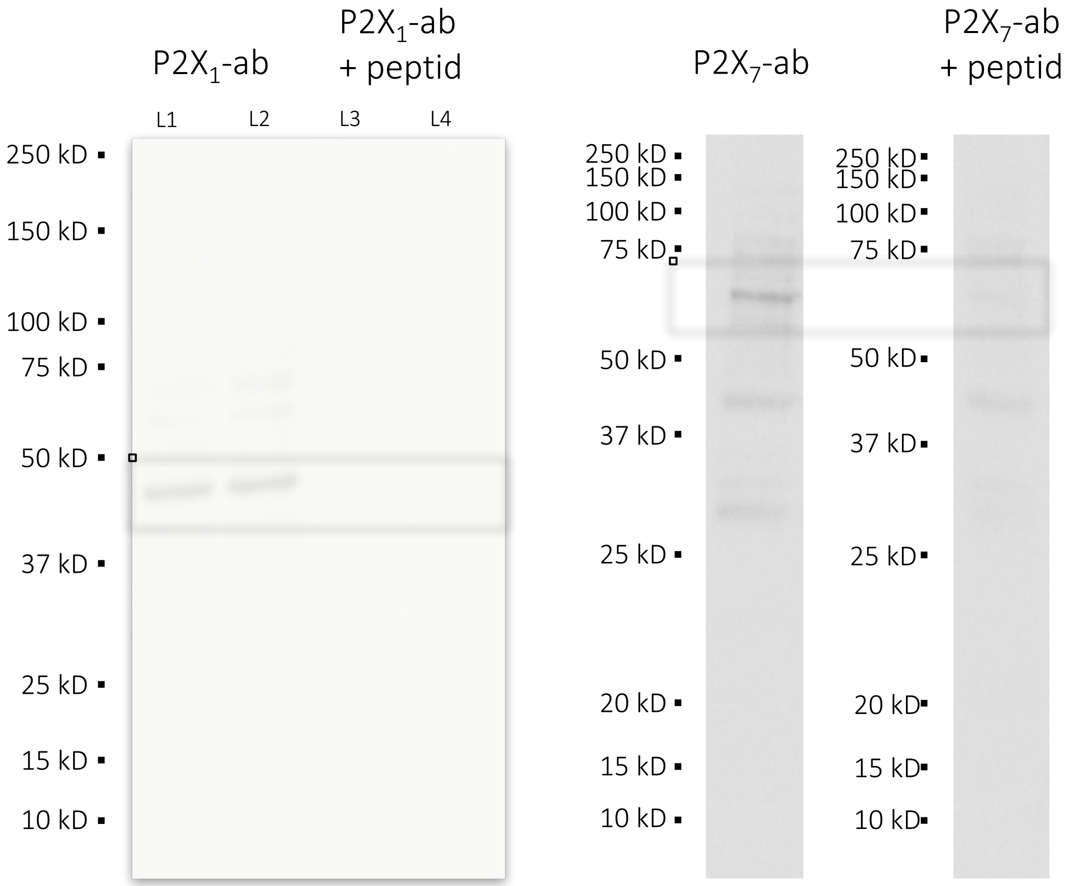

Additional file 2

Immuno-blot with the $P2X_1$ and $P2X_7$ antibodies used for flow cytometric detection of the P2 receptors on the erythrocytes, with or without peptide preadsorption.
